# Multisite evaluation of phenotypic plasticity for specialized metabolites, some involved in carrot quality and disease resistance

**DOI:** 10.1371/journal.pone.0249613

**Published:** 2021-04-02

**Authors:** Wilfried Chevalier, Sitti-Anlati Moussa, Miguel Medeiros Netto Ottoni, Cécile Dubois-Laurent, Sébastien Huet, Christophe Aubert, Elsa Desnoues, Brigitte Navez, Valentine Cottet, Guillaume Chalot, Michel Jost, Laure Barrot, Gerald Freymark, Maarten Uittenbogaard, François Chaniet, Anita Suel, Marie-Hélène Bouvier Merlet, Latifa Hamama, Valérie Le Clerc, Mathilde Briard, Didier Peltier, Emmanuel Geoffriau

**Affiliations:** 1 Institut Agro, Université d’Angers, INRAE, IRHS, SFR 4207 QUASAV, Angers, France; 2 Centre Technique Interprofessionnel des Fruits et Légumes (CTIFL), Paris, France; 3 Vilmorin, Ledenon, France; 4 Rijk Zwaan Breeding BV, Fijnaart, The Netherlands; 5 Rijk Zwaan, Aramon, France; University of Agriculture in Krakow, POLAND

## Abstract

Renewed consumer demand motivates the nutritional and sensory quality improvement of fruits and vegetables. Specialized metabolites being largely involved in nutritional and sensory quality of carrot, a better knowledge of their phenotypic variability is required. A metabolomic approach was used to evaluate phenotypic plasticity level of carrot commercial varieties, over three years and a wide range of cropping environments spread over several geographical areas in France. Seven groups of metabolites have been quantified by HPLC or GC methods: sugars, carotenoids, terpenes, phenolic compounds, phenylpropanoids and polyacetylenes. A large variation in root metabolic profiles was observed, in relation with environment, variety and variety by environment interaction effects in decreasing order of importance. Our results show a clear diversity structuration based on metabolite content. Polyacetylenes, β-pinene and α-carotene were identified mostly as relatively stable varietal markers, exhibiting static stability. Nevertheless, environment effect was substantial for a large part of carrot metabolic profile and various levels of phenotypic plasticity were observed depending on metabolites and varieties. A strong difference of environmental sensitivity between varieties was observed for several compounds, particularly myristicin, 6MM and D-germacrene, known to be involved in responses to biotic and abiotic stress. This work provides useful information about plasticity in the perspective of carrot breeding and production. A balance between constitutive content and environmental sensitivity for key metabolites should be reached for quality improvement in carrot and other vegetables.

## Introduction

Nutritional and sensory quality is an essential attribute of fruits and vegetables [1] with increasing interest from consumers, growers and breeders. Consumed worldwide, carrot is recognized as a healthy vegetable thanks to its substantial content in carbohydrates, dietary fibres and abundance in various specialized metabolites [2]. Carrot is characterized by a particularly high carotenoid accumulation along with significant polyphenol and polyacetylene contents [3]. If carotenoids and polyphenols are widespread in plant kingdom [4,5], some compounds such as polyacetylenes are related to the carrot family *Apiaceae* and other specific botanical families *(Araliaceae* and *Asteraceae)* [6]. These three categories of molecules correspond to health-related compounds exhibiting powerful anti-oxidant activity and preventing cancer development [7–9]. Moreover, some of these compounds are involved in sensory perception [10]. In particular, sweet perception can be influenced by sugar content [11] while terpenes and polyacetylenes may be responsible for bitterness [12] and turpentine-like taste [13]. Actually, several health-related compounds may be involved in bitterness too [12] and a balance between taste and nutritional value must be considered. In an integrative vision of carrot quality improvement, metabolomics is a prevalent method in plant breeding in order to increase nutritional value [14] without compromising taste in carrot.

However, a better knowledge of the determinants of quality related metabolites is still needed to improve and master nutritional and sensorial quality in carrot. Many studies indicate that the genetic basis plays a leading part on nutritional and sensory quality related compounds [15], compared to the environment and growing practices. Recent reviews have assessed the knowledge progress made on the genetic control of major compounds in carrot such as carotenoids [16], terpenes [17], sugars and polyacetylenes [18], highlighting new critical genes. It is well known that environment conditions influence metabolic plant profile [19,20]. Such effects have been quite documented in carrot mainly for carotenoid, sugar, terpene and polyacetylene content [15,20]. Many studies show a higher effect of general climate linked to year effect than specific growing conditions. However, they mostly compare specific factors and involve few varieties, which limits an integrated understanding of quality development in carrot. In particular, the assessment of the respective role of environment and variety depending on the considered metabolite is limited. A better knowledge of these effects for various metabolites in a comparative manner is important to set up a strategy for carrot improvement. There is a lack of multi-criteria approach targeting the main metabolites in carrot involving multiple environments and a larger variety set.

The estimation of phenotypic variability extent for large range of metabolites is determinant in carrot breeding perspectives. Depicted as the organism potential to vary [21], phenotypic variability depends on variety, environment and variety by environment interaction. Dissecting these different factors and evaluating their respective influence on the biochemical content is interesting for prediction purposes. In addition to varietal effect evaluation, a comparative appreciation of the variety by environment interaction effects for metabolite content is necessary for quality assessment and improvement [22,23]. Phenotypic plasticity depicts the ability of a same genotype to express several phenotypes across environments [24]. This plasticity depends on a set of environment perception mechanisms which allow the plant to express appropriate physiological responses to environment fluctuations [25]. Many studies deal about phenotypic plasticity involvement in plant acclimation [26–28]. In particular case, the genetic basis can drive the response intensity to environment constraints and varieties may have a differential of environmental sensitivity. In this way, the study of variety by environment interaction gives information about stability and adaptability potential of varieties [23]. Thus, carrot metabolite profile depends on complex interaction between varieties and locations and according to the studied compound, a variety could be more and less stable. Evaluating and understanding phenotypic plasticity for key metabolites represents a major challenge for producing carrot with high and stable quality. Besides several studies dealing about biochemical variation in carrot root [29–32], none study considering phenotypic plasticity for metabolites related to carrot quality has been reported.

The aim of the present study is to evaluate the level of plasticity for various metabolites involved in carrot quality, in a comprehensive way through a metabolomic approach. In particular, this study aims at identifying metabolite trait stability regarding genotype by environment interaction in order to enhance nutritional and sensory carrot quality. A representative large set of varieties has been evaluated for metabolites content in multiple and contrasted environments over two years. Besides useful information for breeding, the results from this multisite study will allow to appreciate the acclimation capacity of carrot for biochemical quality traits in case of climate changes.

## Materials and methods

### Plant material and experiments locations

This study is based on pluriannual and multilocation trials with randomized three-block designs. A set of 16 varieties representing the current commercial diversity of orange carrot in various types (Table 1) was evaluated the first year over 3 contrasted locations in France (S1 Table). Based on the observed variation on first year, a subset of five varieties was chosen to conduct a multilocation trial on the second and third years with ten locations over four areas in France (S1 Table), leading to evaluation on twenty environments resulting from location and year combination. Trials were conducted during the main cropping season, between mid-June and mid-November depending on locations. Growing conditions were based on local practices. A thinning at two leaves stage was done to control plant density to 50 plants per linear meter. Carrots were harvested around 125 days after sowing and sent to the lab for sampling. For each block, median samples of twenty carrot roots were ground in bulks and stored at -80°C before analysis. Analyses were done on two blocks for the first year and three blocks for subsequent years.

**Table 1:** Studied carrot varieties.

Variety	Code	Type	Source	Genetic structure
Bolero	Bol	Nantes	Vilmorin	Hybrid
Crofton	Cro	Nantes	Rijk Zwaan	Hybrid
De Luc	Luc	Landrace	IRHS/ACO	Population
Dordogne	Dor	Nantes	Syngenta	Hybrid
Extremo	Ext	Nantes	Vilmorin	Hybrid
Maestro	Mae	Nantes	Vilmorin	Hybrid
Morelia	Mor	Nantes	Rijk Zwaan	Hybrid
Nerac	Ner	Nantes	Bejo Zaden	Hybrid
Phoenix	Pho	Nantes	HM Clause	Hybrid
Rodelika	Rod	Colmar	KulturZaat	Population
Romance	Rom	Nantes	Numhems	Hybrid
Sweet-Candle	Swt	Kuroda	Sakata	Hybrid
Verano	Ver	Brasilia	Vilmorin	Hybrid
Vilm4	Vi4	Nantes	Vilmorin	Hybrid
Vilm8	Vi8	Kuroda	Vilmorin	Hybrid
Yukon	Yuk	Nantes	Syngenta	Hybrid

### Metabolic profiling

Seven types of compounds were studied: carbohydrates, acids, carotenoids, polyphenols, 6-methoxymellein, polyacetylenes and volatile compounds with terpenes, sesquiterpenes and phenylpropanoids. All compounds combined, 86 metabolites were quantified.

Carbohydrates and acids were extracted and analysed according to Le Clerc et *al*. (2019) [33] from 10 g of freeze-fresh carrot grinded with 200 mL deionized water. Biochemical compounds were analysed by Acquity UPLC®H-Class system (Waters, Milford, MA, USA) with a photodiode array detector (Acquity PDA eλ) and a refractive index detector (Acquity RI) connected in series. 10 μL of each extract were injected into UPLC column ion-exclusion Rezex ROA-Organic H + 300 mm x 7.8 mm, 8 μm, maintained at 27°C. The mobile phase was composed of 5.10^−4^ N H_2_SO_4_ and elution flow rate was fixed at 0.4 mL/min. Compounds were identified and quantified using the corresponding standards (Sigma-Aldrich, Saint-Quentin-Fallavier, France). Results were expressed in grams per 100 g fresh weight.

Carotenoids were extracted and analysed according to Jourdan et *al*. (2015) [34] and Perrin et *al*. (2016) [20] from 200 mg of frozen carrot samples with duplicate extraction. Samples were analysed by HPLC system (Shimadzu® Prominence I LC-2030 3D) coupled with a photodiode array detector. 20 μL extract were injected into HPLC column YMC® C30 150 mm x 4.6 mm, 3 μm. The mobile phase solution was composed of methanol/acetonitrile/water (84:14:2, v/v/v) with 0.1% BHT (p/v) and triethylamine (v/v) (solution A) and dichloromethane (solution B). The elution flow rate was 0.9 mL/min at 22°C using the following elution gradient: A: 0−12 min, 80−45%; 12−14 min, 45−10%; 14−17 min, 10−80%; 17−23 min, 80%. Compounds were detected at 450 nm and identified according to their retention time and spectral data. They were quantified according to an external standard of β-carotene (Sigma-Aldrich). Results were expressed in β-carotene equivalent in milligrams per 100 g fresh weight.

Polyphenols were extracted in duplicate from 5g of freeze-fresh carrot roots grinded in methanol/water Mq (70:30, v/v) solution. After vortexing, samples were centrifuged at 1912 g during 15 min at 4°C. Supernatant was centrifuged a second time at 1912 g during 15 min at 4°C. Supernatant was then filtered on Acrodisc® 0.2 μm WWPTFE filter. 900 μL of filtrate was pipetted into a 1.5 mL glass vial and 9 μL of Biochanin A (0.08 g/mL) was added as internal standard. Metabolites were analysed by UHPLC system (Waters® Acquity H-class) coupled with a photodiode array detector. Using an auto-sampler set at 5°C, 2 μL of extract was injected into UHPLC column Waters Acquity BEH® C18 100 mm x 2.1 mm, 1.7 μm maintained at 45°C. The mobile phase solution was composed of acetonitrile/water (95:5, v/v) (solution A) and water/acetonitrile (95:5, v/v) (solution B), both with 0.5% acetic acid (v/v). The elution flow rate was 0.3 mL/min with the following gradient: B: 0−4.36 min, 100−80%; 4.36−6.95 min, 80−70%; 6.95−9.26 min, 70−50%; 9.26−11.58 min, 50−25%; 11.58−13.89 min, 25−0%; 13.89−15.00 min, 0−0%; 15.00−16.00 min, 0−100% and equilibration from 16.00–22.00 min, 100% B. UHPLC system was coupled with a Time of Flight-Mass spectrometer (Waters® Xevo G-2S) equipped with an electrospray ionization source in negative mode. The ion transfer capillary temperature was set at 350°C and needle voltage at 2.5 kV. Nitrogen was used as nebulizing gas with flow rate 25L/h. The spectra were acquired within mass ranging from 50−1200 Da, with no collision in low energy and ramp collision from 15 to 40 in high energy. Data acquisition and processing were performed using MassLynxTM and TargetLynxTM software. Results were expressed in Biochanin A equivalent in nanograms per g fresh weight.

Volatile compounds, 6-methoxymellein and polyacetylenes were extracted and analysed according to Le Clerc et *al*. (2019) [33] from 10 g of freeze-fresh carrot grinded samples. Extractions were triplicated. The same extract was used for volatile compounds, 6-methoxymellein and polyacetylenes analysis. Volatile compounds were analysed by GC-FID and GC-MS systems described by Aubert et *al*. (2017) [35] from 1 μL of sample injected into GC systems. Compounds were identified according to their retention time and mass spectrum data and results were in ppb 2-octanol equivalent. The analysis of 6-methoxymellein and polyacetylenes requires the addition of 1mL of acetonitrile to 100 μL of extract and was carried out on UHPLC system (Waters® Acquity UPLC) coupled with a photodiode array detector using a Perkin Altus BEH C18 column 50 mm x 2.1 mm, 1.7 μm as described in Le Clerc et *al*. (2019) [33]. The mobile phase solution was composed of water (solution A) and acetonitrile (solution B). The elution flow rate was 0.4 mL/min with the following elution gradient: B: 0–1 min, isocratic 20%; 1–10 min, linear 20–50%; 10–30 min, linear 50–95%; 30–35 min, isocratic 95%. Compounds were identified according to their retention time and spectral data. Results were expressed in ppm 4-chlrorobenzophenone equivalent. The concentrations should be considered as relative data because recovery after extraction and calibration factors related to the standards were not determined.

### Statistical analysis

All statistical analyses were computed with R version 3.5.1. Permutational multivariate analysis of variance was performed by PERMANOVA on centered-scaled metabolites content. PERMANOVA was computed from Bray-Curtis dissimilarity matrix and 9999 permutations, using R vegan 2.5–6 package. In order to better understand which factor most influences the global biochemical content, a PCA was computed on all 86 biochemical compounds content scaled from means of two agronomic replications, using R FactoMineR 2.3 package. PCA area was computed according to variety coordinates on three principal components and expressed as mixt product: Area (*ALS*) = $\frac{1}{2}[\underset{AL}{\to },\underset{AS}{\to }]=\frac{1}{2}$ det ($\underset{AL}{\to },\underset{AS}{\to }$), where three points A, L, S represent respectively each location Ac, Lv and Sa. Variety areas are represented in a 3-dimensional space thanks to geogebra software using PCA coordinates of each condition on 3 first principal components.

Random forest algorithm was used to discriminate varieties according to their metabolic profiles. Data were pre-processed by centering and scaling, and random forest was computed using Rfpermute 2.1.81 package, using 500 trees and 2000 permutations. Out Of Bag (OOB) method was used to estimate the model prediction error. Selection of biochemical compounds was carried out based on variable importance in classification according to Mean Decrease Gini criteria and Mean Decrease Gini (*p*.*value < 0*.*05*). Variety effect on identified compounds was tested thanks to one-way ANOVA, or Kruskal-Wallis test in case where parametric test conditions were not met. Post hoc tests were realized, with respectively Tukey HSD test after ANOVA and Conover test after Kruskal-Wallis, in order to determine which variety accumulates in higher proportion a given compound. To be able to compare the variation for metabolites of interest, the Environmental Variation Coefficient was computed according to $Cv=\frac{Sdvar}{Yvar}$, where Sdvar is standard deviation of the variety for a given compound and Yvar is biochemical content average of the variety for the same compound [36]. Hierarchical clustering was computed according to Euclidean distances and dendrogram was built according to Ward method based on the highest axes inertia.

Compounds accumulated by all varieties were used to compute plasticity amplitude based on the difference between the highest and the lowest value for each variety. Studied in 2017–2018 over 20 environments, the theoretical amplitude was computed based on the sum of all compound amplitudes for varieties (Sci) and the sum of all varieties amplitudes for this compound (Srj) on the total sum of amplitudes (St): theoretical amplitude = $\frac{Sci\;x\;Srj}{St}$. The difference between observed and theoretical amplitudes was measured by standardized Pearson residuals. For compounds with high absolute Pearson residuals sum, a linear regression was realized for each variety using biochemical content on the average biochemical content of all varieties for each location [37]. The environmental sensitivity of a variety is depicted by bi coefficient and illustrates the variety response intensity to the environment. Whereas ecovalence [38] depicts variety contribution to SCE of variety by environment interaction term. For variety with strong ecovalence, an additive main effects and multiplicative interaction (AMMI) model [39] was computed using R stability 0.5.0 package, in order to identify environments where variety performs best.

## Results

### Extent of biochemical content variation

The biochemical variation was first evaluated on 16 varieties over three environments for 86 compounds. Metabolite profiles were very significantly influenced by variety, location and variety with location interaction (*p*.*value <0*.*001*) (Table 2). In comparing mean squares, the location effect explained the most important variation part with 58.87%, followed by variety effect with 35.62% and genotype by environment effect with 4.2%. With only three environments, variety by location interaction represented a substantial level with 11.79% of variety main effect. Overall, the impact of locations was higher than varieties, which confirms the predominant effect of environment on metabolite content in carrot.

**Table 2:** PERMANOVA results based on 86 metabolites analyzed using Bray-Curtis dissimilarity index.

Factors	DF	SumsOfSqs	MeanSqs	R^2^	F.Model	Pr (>F)
**Locations**	2	0.7479	0.3740 *(58*.*87%)*	0.14063	45.8619	1.00E-04***
**Varieties**	15	3.394	0.2263 *(35*.*62%)*	0.6382	27.7502	1.00E-04***
**Variety by location interaction**	30	0.7848	0.0267 *(4*.*2%)*	0.14758	3.2085	1.00E-04***
**Residuals**	48	0.3914	0.0082 *(1*.*31%)*	0.07359		
**Total**	95	5.3181		1		

With DF: Degrees of freedom; SumsOfSqs: Sums of squares; MeanSqs: Mean of squares; R^2^: R-squared; F.Model: F-test value for model and *Pr (>F)*: p-value.

**Fig 1:**
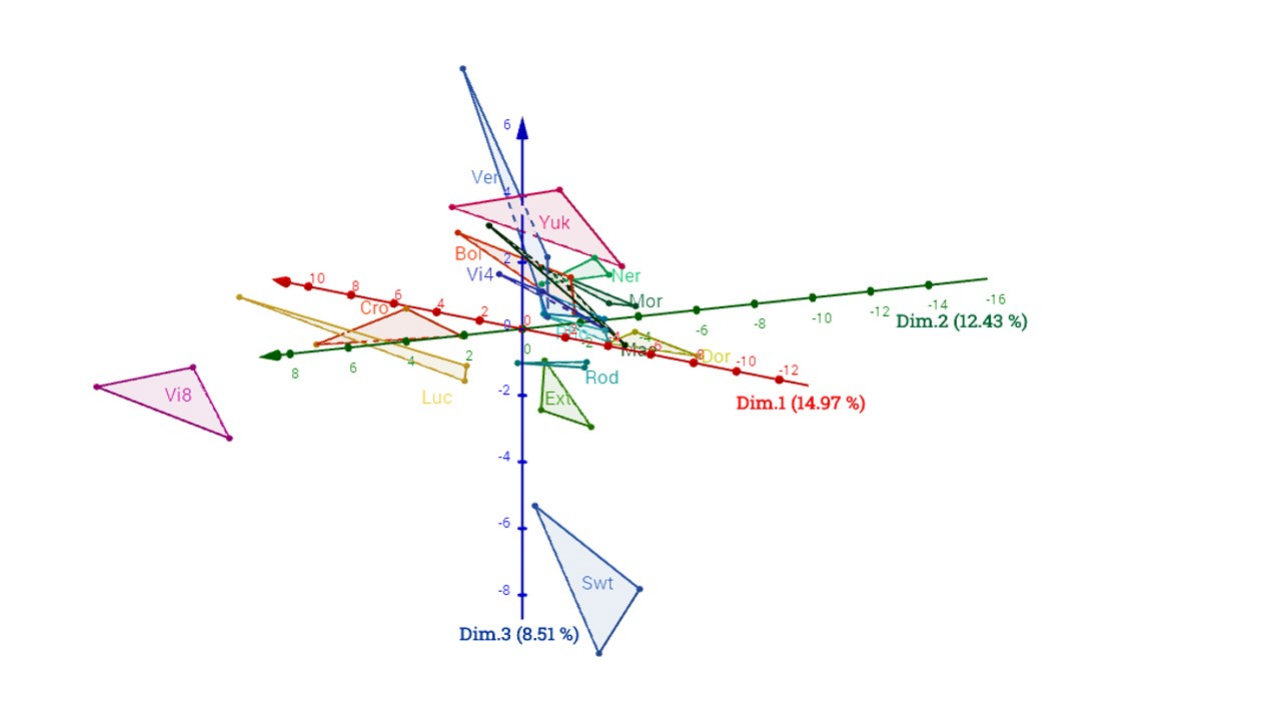
Distribution of varieties by Principal Component Analysis (PCA) based on 86 metabolites content on 3 environments. The 3 first components explain 35.89% of total inertia. For variety code, see Table 1.

The variation distribution was represented by principal components analysis (PCA). The first three PCA components explained only 35.89% of total variance with respectively 14.97%, 12.43% and 8.51% (Fig 1), due to the high number of variables and various factor effects. Overall the PCA shows distinction between varieties. However, the first component shows more particularly the environment effect on metabolite profiles and in less extent the variety effect, in accordance with the variance analysis. Second and third components explain more especially the varietal effect (Fig 1). In fact, few varieties (Vi8, Cro and Luc) are separated from the other varieties according to the second component and particularly one variety (Swt) according to the third one. Thus, the PCA analysis shows that Vi8 and Swt varieties seem to be biochemically different from the other varieties and from one another, which can be explained by their Kuroda background.

A differential effect of locations on varieties is highlighted by area computation on PCA components (Table 3). The PCA areas ranged from 0.4 and 18.4 for unweighted areas, and in a similar way from 0.44 and 20.32 weighted areas. Very high differences between varieties were observed with differences up to 45 times between the less variable and the most variable varieties for metabolite content. The extend of variation is the lowest for Rod, Mor, Ner and Pho varieties, and the highest for Vi8, Mae, Yuk and Cro, which illustrates differential responses of these varieties depending on the environments for metabolite content.

**Table 3:** Extend of variety variation as shown by PCA areas.

Variety	Vi8	Mae	Yuk	Cro	Vi4	Swt	Bol	Luc	Rom	Dor	Ext	Ver	Pho	Ner	Mor	Rod
**Area**	18.40	16.45	15.16	11.36	10.81	9.51	5.65	5.39	5.15	4.79	4.18	3.78	2.74	2.72	0.95	0.40
**Weighted area**	20.32	18.21	16.75	12.40	11.86	10.68	6.33	5.95	5.60	5.23	4.60	4.21	3.02	2.96	1.07	0.44

Areas were computed according to the three first components of PCA. Weighted areas depict a ponderation by component inertia.

Even if results show a substantial effect of environment on metabolic profiles, the genetic background seems to play a leading part both on variety specific metabolite accumulation or metabolite content variation. Besides clear-cut discrimination of Kuroda type from other varieties, a differential effect of locations on varieties is highlighted.

### Biochemical compounds involvement in variety discrimination

**Fig 2:**
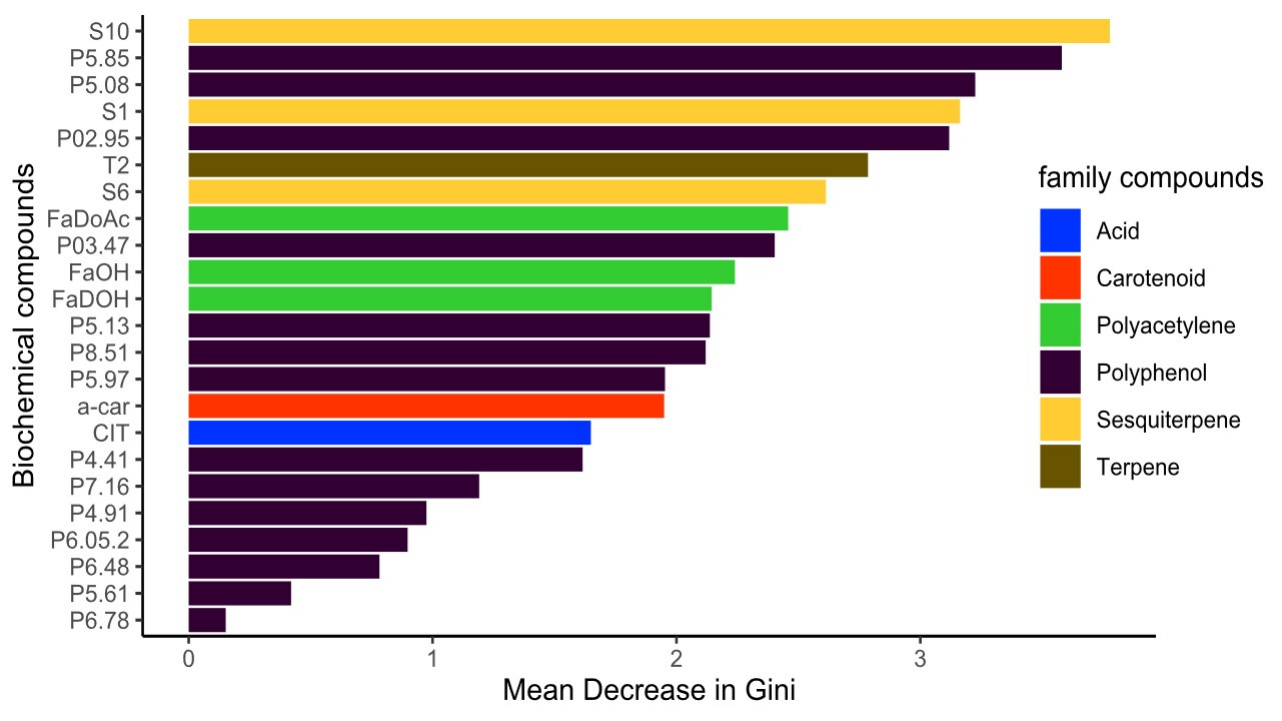
Significant variables in decreasing importance for varietal clustering based on Random forest method. For compound identification, see Tables 5 and S4 for polyphenol molecules characteristics.

PCA is actually an unsupervised method which lacks performance to identify the role of specific metabolites in differences between varieties and therefore to characterize variety variation for biochemical content. Random forest model was used to determine discriminant metabolites in variety characterization. Random forest model showed a high level of prediction, with only 1.04% of prediction error, only one individual being misclassified out of 96 individuals (S1 Fig). Over 86 analysed metabolites, 24 displayed a significant and high effect on variety clustering (Fig 2) based on Mean Decrease in Gini criteria (*p*.*value <0*.*05*). Polyphenol compounds were the most represented ones, with 14 compounds. Then polyacetylenes and sesquiterpenes with three compounds respectively. Finally, citrate, α-carotene and the terpene β-pinene (T2) were also involved in variety clustering. Noticeably, important quality criteria such as carbohydrates and β-carotene content were not selected and therefore not the most discriminating factors between varieties.

**Fig 3:**
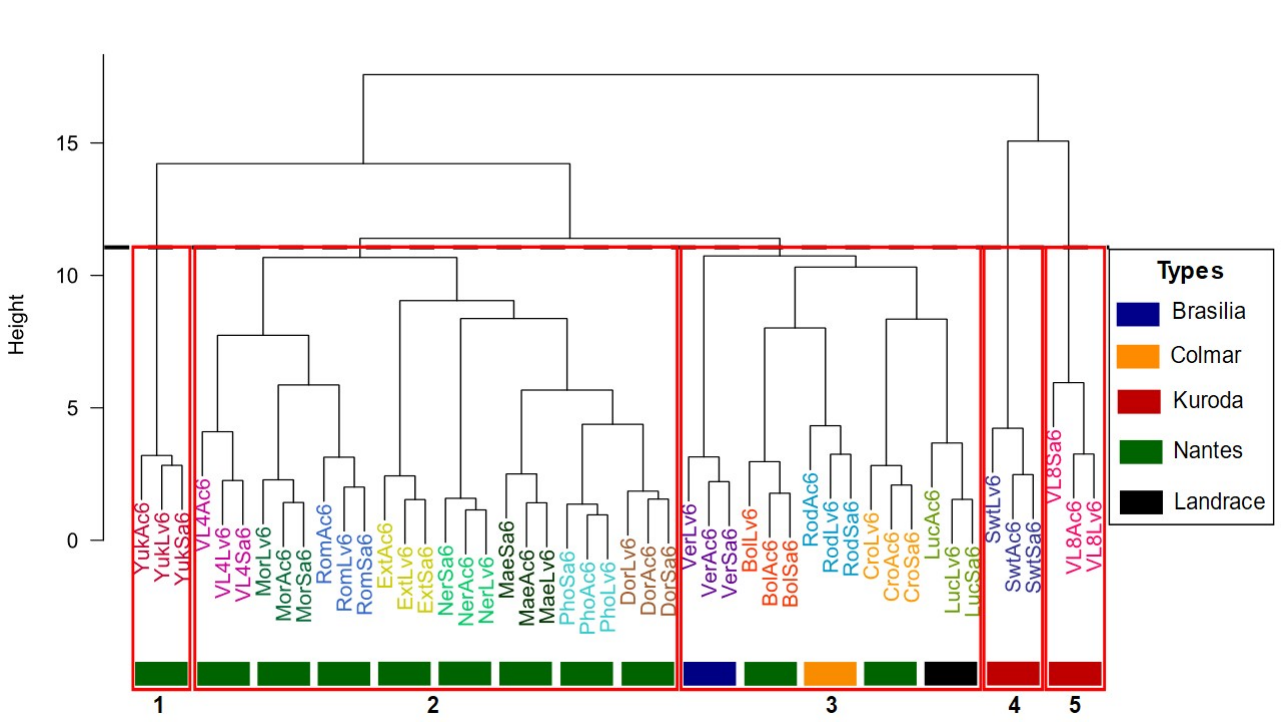
Variety hierarchical clustering on the 20 most discriminating metabolites according to Random forest model over 3 environments. Distances are computed according to euclidean distance and dendogram is built according to ward method. Colored rectangle at the bottom of the dendogram highlights the carrot type.

Based on the 20 most discriminating compounds (Mean Decrease in Gini >1), hierarchical clustering on Euclidean distance matrix scaled block mean shows an effective separation between varieties (Fig 3). Moreover, the clustering is related to variety type. The first cluster is only composed of one variety, an early Nantes carrot type. The second cluster is composed of eight varieties from the same type, Nantes type. The third one is composed of five varieties but from four carrot types. The fourth and fifth clusters are only composed of one variety, both of Kuroda type. These results show a clear structuration of carrot diversity based on metabolite content in relation with carrot types.

**Fig 4:**
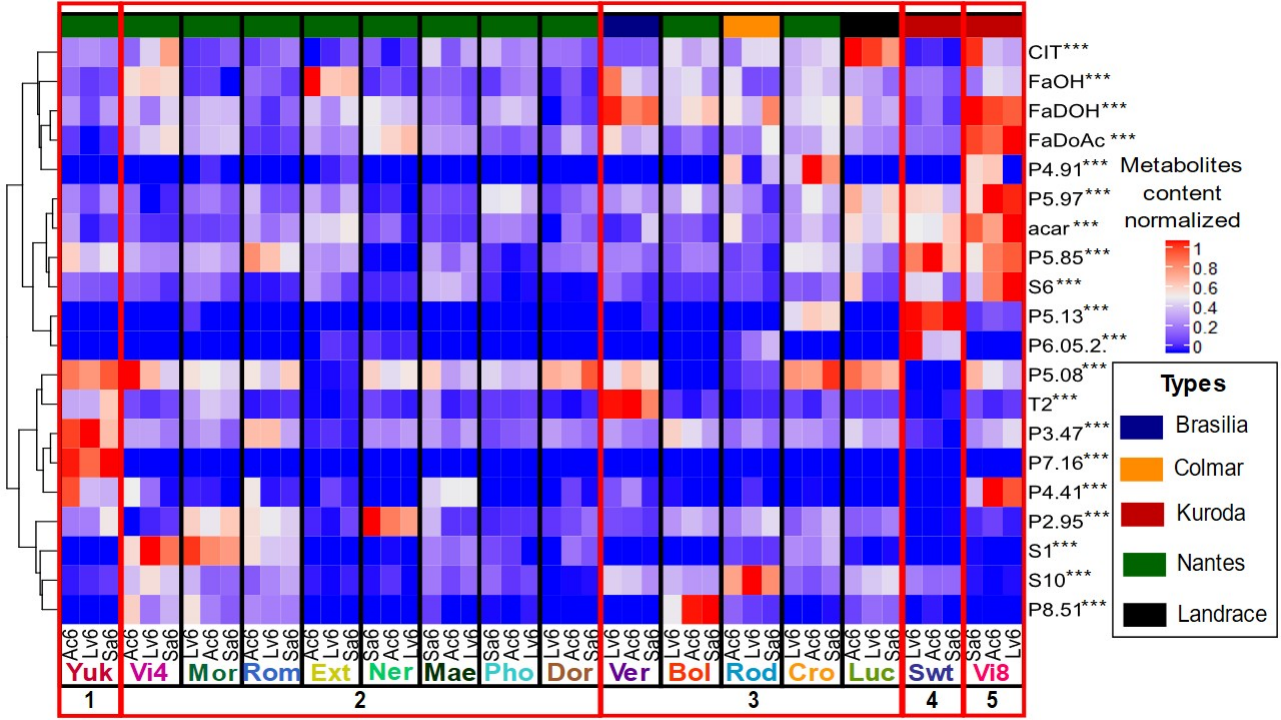
Variety heatmap on 20 metabolites selected according to Random forest model. Couples variety/location are in column and metabolites in lines. In red, high content compounds and in blue, low content compounds. Compounds codes are presented in Table 5 (P compounds are unknown polyphenols. For polyphenol information, see S4 Table).

In a comprehensive way, some compounds can characterize specifically varieties, as shown by the heatmap based on relative content and table content (Fig 4, S2 Table). This is particularly the case for P7.16 compound which is accumulated by only one variety from cluster 1. For the corresponding variety and compound, environment has moreover a low influence on content (CV = 8.02%). Quantitatively, P3.47 and P5.08 are also discriminating, with P4.47 being highly accumulated in a single variety and P5.08 in a few varieties, compared to others. However, if P5.08 proportion is considered as relatively stable (CV = 9.11%), P3.47 content is variable and significantly influenced by environment (CV = 22.66%). A combination of metabolites can characterize a variety. If P5.08 is not accumulated in cluster 4 variety, P5.13 and P6.05.2 are accumulated in higher proportion. P5.13 proportion is considered as stable (CV = 3.00%) whereas P6.05.2 content, as unstable (CV = 58.14%). In this way, P5.08, by its absence, and P5.13, by its stable high proportion, are characteristic from this variety. In a similar way, three compounds allow the discrimination of cluster 5 Vi8 variety compared to the other varieties. If several compounds are accumulated in higher proportion by this variety, three metabolites content are less influenced by environment i.e. polyacetylenes falcarindiol (FaDOH) (CV = 5.21%), falcarindiol-acetate (FaDOAc) (CV = 6.25%) and α-carotene (acar) (CV = 11.26%).

Interestingly, overall low accumulation of metabolites characterizes the Nantes type varieties in cluster 2, which can explain the clustering from other Nantes type varieties (Figs 3 and 4). We observe three metabolic profiles based on specific compounds. A higher content in b-elemene (S1) is characteristic for Mor and Vi4 with a moderate stability pattern of 28.27% and 14.23% CV respectively. FaOH and P2.95 discriminate respectively Ext and Ner from other varieties with substantial environment effect (CV = 23.15% and 16.63% respectively). P5.08 is highly accumulated in one variety (Dor) compared to other varieties in this cluster but similarly to 2 other Nantes type varieties from cluster 1 and 3.

Several compounds allow discrimination among varieties in cluster 3. β-pinene (T2) and FaDOH characterize Ver variety and biochemical content is stable across environments with CV = 11.92% and 8.77%, respectively. The high level of P8.51 and α-bisabolene (S10) discriminate respectively Bol and Rod from all other varieties, even if the content may vary depending on the environment (CV = 35.26% and 18.53% respectively). Interestingly, the landrace variety Luc is distinguished by the highest and stable content in citric acid (CIT, CV 12.23%).

Our results indicate that polyphenol compounds seem to play a major role in variety differentiation and deserve more attention in carrot. Polyacetylenes and terpenoids are largely involved in variety discrimination. Among terpenoids, β-pinene (T2) is the only terpene characterizing one variety from others whereas two sesquiterpenes compounds, b-elemene (S1) and α-bisabolene (S10), seem to be characteristic of a few varieties. The polyacetylenes, mainly the falcarindiol forms, are involved in several varieties. In a few cases only, the combination of compounds is useful to discriminate varieties.

### Evaluation of phenotypic plasticity

Considering the 51 compounds accumulated by all varieties, phenotypic plasticity was first assessed on the first-year results by measuring global amplitude response over 3 environments of diversity set varieties (Table 4). The amplitude means ranged from 1.34 to 2.04 and standard deviation from 0.87 to 1.33, suggesting different phenotypic plasticity patterns between studied varieties. Congruently with above observations, phenotypic plasticity level is the lowest for cluster 2 varieties, with low standard deviation compared to cluster 3 varieties. Plasticity level is the lowest for Dor variety whereas Mae exhibits a particular pattern, with high amplitude mean and low standard deviation. Single variety clusters show various plasticity patterns. If cluster 5 variety Vi8 has the most important phenotypic plasticity level with the highest amplitude mean and standard deviation (2.04 and 1.28 respectively), cluster 1 variety Yuk and cluster 4 variety Swt present moderate and low plasticity respectively. It suggests that clustering varieties according to their plasticity level in addition to clustering based on variety specific compounds accumulation may a better way to characterize varieties.

**Table 4:** Variety amplitude based on 51 metabolites on three environments.

	Vi8	Luc	Cro	Mae	Ver	Yuk	Bol	Rod	Vi4	Pho	Mor	Ner	Swt	Ext	Rom	Dor
**Sum of amplitudes**	103.8	102.2	101.9	95.7	94.2	93.1	92.9	91.7	89.4	75.4	75.2	73.2	72.9	72.8	72.0	68.2
**Mean of amplitudes**	2.04	2.00	2.00	1.88	1.85	1.83	1.82	1.80	1.75	1.48	1.47	1.44	1.43	1.43	1.41	1.34
**Standard deviation**	1.28	1.24	1.13	0.91	1.21	1.19	1.05	1.33	1.02	0.93	1.01	0.87	1.23	1.07	1.06	0.90

In green, cluster 1 variety; red, cluster 2 varieties; blue, cluster 3 varieties; orange, cluster 4 variety and purple, cluster 5 variety (see Figs 3 and 4).

In order to heighten phenotypic plasticity evaluation of compounds, a subset of five varieties (Vi8, Cro, Mae, Ver and Dor), differing for the general plasticity level and carrot type, was cultivated on a large set of 20 environments (2 years and 10 locations). Pearson residuals on differences between observed and theoretical amplitudes were used to determine compounds with stronger differential of plasticity. The absolute Pearson residuals sum ranged from 4.14 to 0.96 (Table 5). Compounds with higher phenotypic plasticity differential are phenylpropanoids (PP2 and PP1), terpenes (T2), sesquiterpenes (S6, S10, S1) and the phenolic compound 6-methoxymellein. Among polyacetylenes, phenotypic plasticity differential is higher for falcarindiol acetate (FaDOAc) than for falcarindiol (FaDOH) and falcarinol (FaOH). Even if there is a substantial phenotypic plasticity for carbohydrates (sucrose, glucose and fructose) and β-carotene, differential of plasticity is low for these compounds. This is congruent with above results on a large set of varieties.

**Table 5:** Absolute values of Pearson standardized residuals of biochemical compounds on 5 carrot varieties and 20 environments.

Code	Compound	Sum	Mean	Standard deviation
**PP2**	Myristicin	4.14	0.83	1.01
**S6**	D-germacrene	4.13	0.83	0.95
**T2**	β-pinene	4.12	0.82	1.18
**S10**	α-bisabolene	4.12	0.82	1.12
**PP1**	Elemicin	3.90	0.78	0.96
**6MM**	6-methoxymellein	3.71	0.74	0.88
**S1**	β-elemene	3.67	0.73	0.88
**T15**	cis-β-ocimene	3.59	0.72	0.99
**T3**	sabinene	3.31	0.66	0.94
**T5**	β-myrcene	3.08	0.62	0.71
**T18**	Unknown	3.06	0.61	0.77
**T1**	α-pinene	2.99	0.60	0.76
**FaDoAc**	falcarindiol acetate	2.88	0.58	0.74
**S5**	(E)-β-farnesene	2.77	0.55	0.69
**T16**	trans-β-ocimene	2.64	0.53	0.72
**S2**	β-caryophyllene	2.62	0.52	0.65
**S11**	bisabolol	2.53	0.51	0.61
**acar**	α-carotene	2.37	0.47	0.65
**T17**	unknown	2.36	0.47	0.64
**S4**	α-humulene	2.21	0.44	0.61
**S9**	γ-bisabolene	2.05	0.41	0.55
**FaDOH**	falcarindiol	1.98	0.40	0.50
**T14**	β-phellandrene	1.95	0.39	0.56
**FaOH**	falcarinol	1.94	0.39	0.45
**SAC**	sucrose	1.93	0.39	0.45
**T7**	γ terpinene	1.79	0.36	0.44
**S13**	unknown	1.78	0.36	0.49
**T8**	p cymene	1.77	0.35	0.42
**CIT**	Citric acid	1.76	0.35	0.44
**T6**	limonene	1.72	0.34	0.43
**lut**	lutein	1.60	0.32	0.47
**S7**	β-bisabolene	1.59	0.32	0.45
**GLU**	glucose	1.52	0.30	0.35
**T9**	α-terpinolene	1.48	0.30	0.40
**T4**	α-phellandrene	1.41	0.28	0.40
**Bcar**	β-carotene	1.39	0.28	0.38
**MAL**	Malic acid	1.33	0.27	0.41
**FRU**	fructose	0.97	0.19	0.27
**S3**	β-farnesene	0.96	0.19	0.24

**Fig 5:**
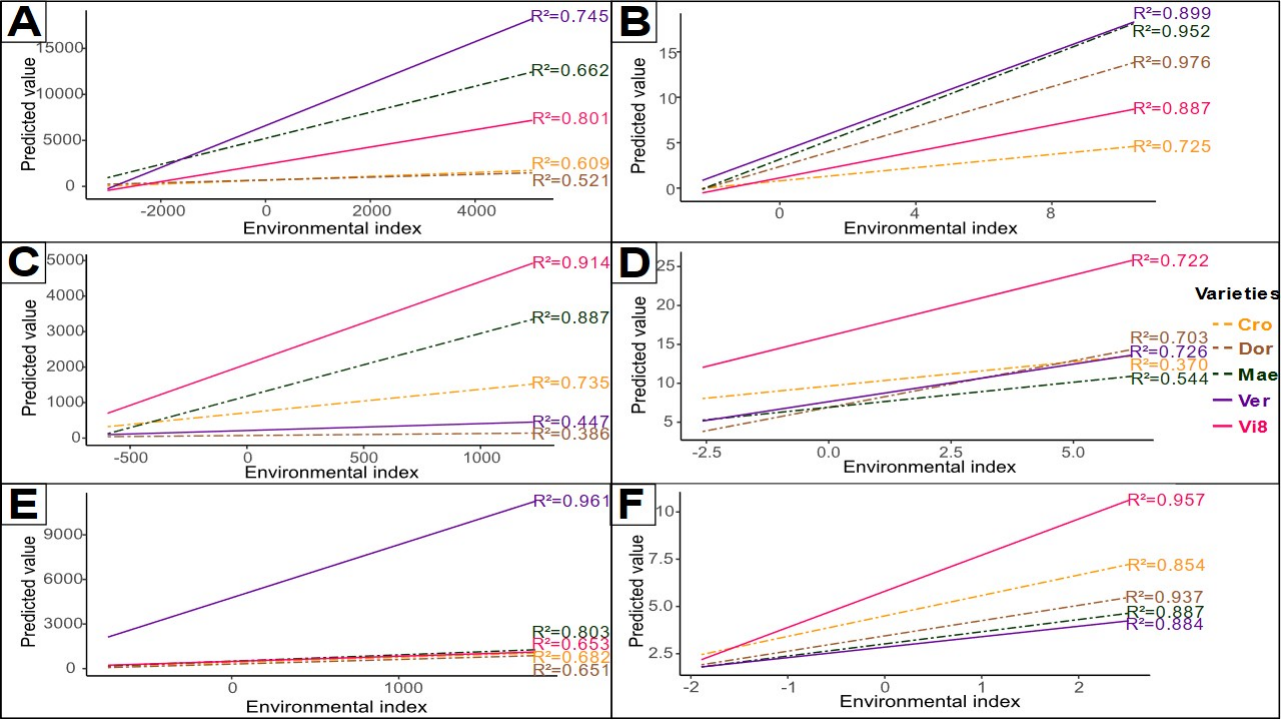
Environmental sensitivity of five varieties for six compounds over 20 environments. (A) PP2 myristicin, (B) 6MM 6-methoxymellein, (C) S6 D-germacrene, (D) FaDOAc falcarindiol acetate, (E) T2 β-pinene and (F) acar α-carotene. X-axis represents environmental index for compounds synthesis all varieties combined, and Y-axis level of predicted synthesis for each variety. Slope is traced according to regression coefficient bi (S3 Table).

Phenotypic plasticity pattern was characterized on a set of six key quality compounds chosen according to literature and differential plasticity level of varieties. Selected compounds presented a sum of absolute Pearson residuals (Table 5) above the median (2.21), ranging from 4.14 to 2.37. For a given compound, an important regression slope, corresponding to a high bi coefficient (S3 Table), depicts high environmental sensitivity of a variety (Fig 5). Whereas strong ecovalence term reveals an atypical variety behaviour face to the environments compared to other varieties (S3 Table). According to selected compound, a variety with high environmental sensitivity concomitantly has a strong ecovalence term. Globally, exotic varieties (Ver and Vi8) are the most sensitive to the environment, with respectively three compounds for which they exhibit a strong environmental sensitivity. Considering compounds in a more singular way, β-pinene (T2) has a particular pattern with a strong influence of environment for only one variety (Ver) whereas environment has limited effect on other varieties (Fig 5E). A strong differential of environmental sensitivity can be observed for Myristicin (PP2) (Fig 5A) and D-germacrene (S6) (Fig 5C). Noticeably, Dor variety has extremely low levels of environmental sensitivity for these two compounds accumulation. Differential of environmental sensitivity is less pronounced for 6-methoxymellein (6MM) (Fig 5B) and α-carotene (acar) (Fig 5F) accumulation, with moderate to high influence of environment in compound accumulation, according to variety. Finally, a particular pattern can be observed for falcarindiol-acetate (FaDoAc) (Fig 5D) with substantial effect of environment for several varieties and rank inversion.

**Fig 6:**
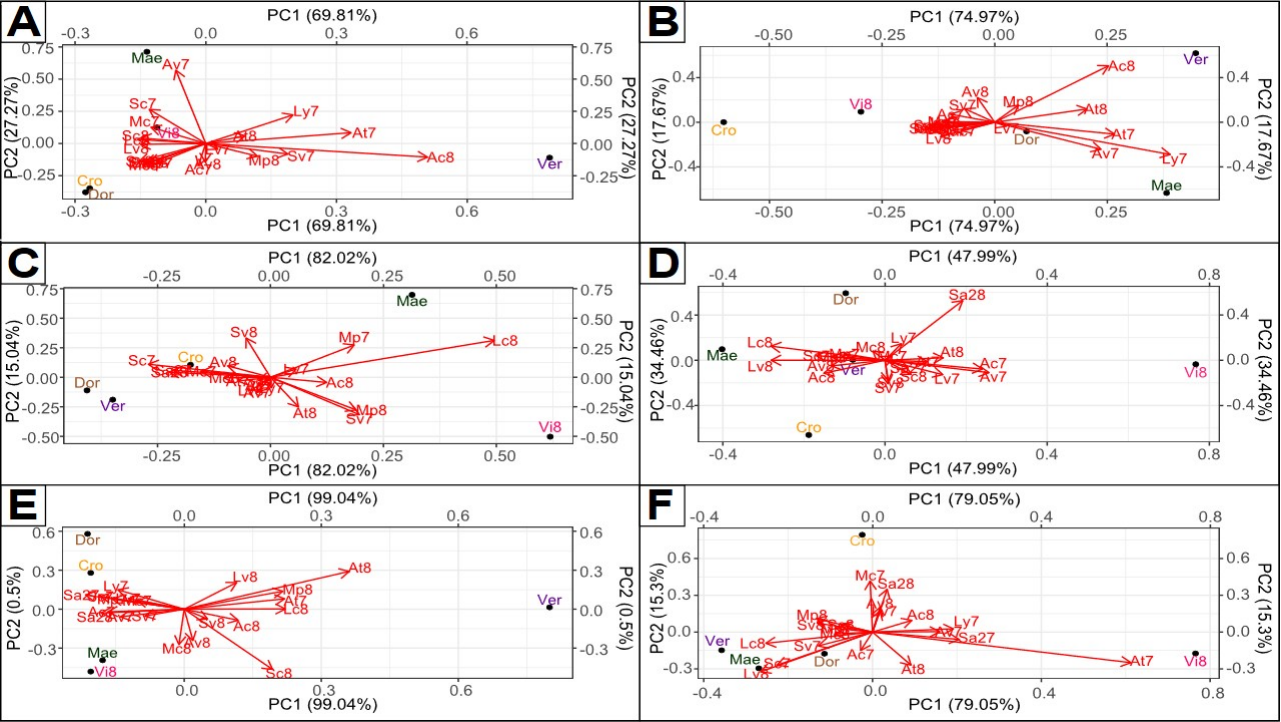
Identification of favorable environments for five varieties and six compounds exhibiting phenotypic plasticity, based on the AMMI model. (A) PP2 myristicin, (B) 6MM 6-methoxymellein, (C) S6 D-germacrene, (D) FaDOAc falcarindiol acetate, (E) T2 β-pinene and (F) acar α-carotene. Varieties near to the center of biplot have no specific location to enhance metabolite content, contrary to the most external varieties which have predilection location for accumulation. Thus, a variety very close to environment vector highlights high metabolite enhancement of this variety on this environment compared to other varieties. Besides, varieties on right border are varieties with high accumulation potential.

As described above, accumulation for some metabolites can be highly dependent on genotype by environment interaction. It justifies a more comprehensive investigation in order to identify environments enhancing metabolite accumulation. We used AMMI model as a complementary tool to characterize metabolite patterns depending on accumulation promoting environments. Several patterns can be observed for the six studied compounds (Fig 6). For falcarindiol acetate (FaDOAc) (Fig 6D) and α-carotene (acar) (Fig 6F), especially one environment has a large influence on compound accumulation, as shown by the large vector compared to others. In this case, no specific advantage is observed for most environment-genotype combinations which indicates that limited progress is expected and no specific effort is overall needed for these compounds. However, only a very few combinations need to be avoided (to limit bitterness due to falcarindiol acetate for example) or promoted (for enhancing α-carotene level). Pattern is intermediate for D-germacrene (S6) (Fig 6C) with a large effect of one environment and substantial effect of several other environments. In this case, the choice of environments is important to increase the level of this health benefit compound by expressing the synthesis potential of certain varieties (Mae and Vi8 as exemplified in Fig 6C). Interestingly, a same pattern can be observed between myristicin (PP2) (Fig 6A) and 6-methoxymellein (6MM) (Fig 6B) accumulation. The same environments, mainly from center-west geographical area, and the same varieties (Mae and Ver) are favourable for the accumulation of both compounds. Finally, a specific pattern is observed for β-pinene (T2) accumulation (Fig 6E) with favourable 2018 related environments in combination with Ver variety. Main year effect can be explained by a warmer and drier year in 2018 compared to 2017 (S1 Table). As shown by our AMMI results, metabolite content prediction could be envisaged for these three bitterness related compounds, with patterns linked to production areas or year conditions.

The phenotypic plasticity level differs between compounds and the extent of plasticity varies depending on varieties. Compounds with the higher differential of phenotypic plasticity level belong to terpenoids and phenolic families. The study of phenotypic plasticity must be done on a metabolite-by-metabolite basis. The use of varietal accumulation amplitude occurs as an effective approach to select compounds under both genetics and environmental determinism, facilitating such approaches. Considering environmental sensitivity for metabolites of interest is essential for prediction purposes.

## Discussion

Metabolic profiling was used as an efficient approach to grasp phenotypic variability for various metabolites involved in carrot quality. Our results show the overall major effect of variety on carrot metabolic content, whether due to specific metabolite synthesis or differential of phenotypic plasticity level between variety according to studied compound. Two concepts are largely opposed to evaluate varietal stability: static stability and dynamic stability [40]. Considering stability concepts in conjunction with large metabolic profiling, our study is the first which integrates phenotypic plasticity to varietal effect in order to characterize and promote whole carrot quality potential. To our knowledge, no study deals about stability and adaptability of varieties on key metabolites content, all fruits and vegetables considered.

### Stability and adaptability concepts applied to metabolic profiling

Stability concept allows to assess varieties fluctuations whereas adaptability considers genotype by environment interaction to depict variety ability responding positively to the environment [23]. Widely used in agronomy, these two concepts are more applied to biomass yields. Our work highlights two main difficulties for the application of this type of concept to metabolomic data. Contrary to yield analysis which is univariate, the first hurdle encountered in metabolomic studies is the large number of variables, which requires sorting and prioritization to better study effects on involved compounds. Recognized thanks to its predictive accuracy as a classification method for complex traits genome-wide prediction [41], we used Random forest algorithm which is particularly adapted in metabolomic for biomarker identification [42–44]. The use of several contrasted growing locations allows to introduce environmental source of variation which is essential to assess variety stability for a given trait but may degrade algorithm predictive accuracy used for varietal marker characterization. Moreover, our study and Sampaio works [19] show a substantial effect of environment on plant metabolic profiles. Since the use of Gini index only can overvalue feature importance in classification [45], we used the permutation of variables performed in Rfpermute to estimate importance metrics significance and highlight varietal markers with low environmental sensitivity. Noticeably, most of biochemicals with substantial Gini index and non-significant mean decrease in gini p.value are compounds where variety by environment interaction is considerable. Contrary to biomass production, a second difficulty lies in the accumulation of metabolites with very different proportions and scale units according to the compound family. Thus, comparison of varietal stability for different compounds could be harder to assess in an integrative metabolomic analysis. We found appropriate to compare metabolite variation content due to environment in relation with varietal mean content, to compare metabolites accumulation which each other. In this way, coefficient of variation [36] is preferred to compare biochemical content variation when environmental variance is widely used to assess yield stability [40].

Besides issues related to data mining for stability study applied to metabolic profiling, biomass and metabolite accumulation are not influenced in the same manner. Metabolome is strongly dynamic and plant metabolic profiles depend on various level of regulation. Kooke and Keurentjes review [46] highlights four large dimensions contributing to metabolome diversity: a temporal regulation, a spatial regulation, an environmental regulation and finally genetic regulation. In this way, prediction purpose for metabolite content may occur harder to assess due to large range of regulation levels involved and transitory states of several metabolites. Whatever the level of variety plasticity differential for metabolites highlighted in our study, the environmental sensitivity range is higher for biochemical content compared to barley and durum wheat yield [47,48], yield resulting from plant growth and development with a possible buffer effect. Considering both static and dynamic stability of varieties when studying metabolite content is determinant in quality elaboration and varietal creation to guarantee nutritional and sensorial value in carrots.

### Assessment of constitutive ability of varieties through stability concept

Our study highlighted 20 metabolites largely involved in variety discrimination under low environmental determinism, showing the constitutive ability differential of varieties to accumulate key metabolites. Corresponding varieties can be considered as statically stable for given compounds. This concept is related to biological construct of homeostasis and the corresponding index is interesting for variety selection as it is known to be repeatable in time [49]. For these highlighted metabolites, breeding effort would be efficient to promote or reduce their content.

Among highlighted varietal markers, polyacetylenes and α-carotene have nutritional value and are recognized as health benefit molecules with large application in the medical field [8,9,50–54]. A particular pattern was observed for α-carotene between varieties. High proportion is depicted for two varieties belonging to the same carrot type Kuroda, suggesting that genetic background is involved in α-carotene content enhancement. Carotenoid content depends on different regulation scale with regulation on anabolism, catabolism/degradation and finally at chromoplast level as source to sink [55]. Previous work highlighted the leading part of deficient carotene hydroxylase CYP97A3 allele in carrot α-carotene accumulation [56], while effective allele is involved in metabolization of α-carotene in lutein [57]. Lutein to α-carotene ratio being the lowest for one of two kuroda, and relatively low for the second compared to the other varieties, defective allele for CYP97A3 could explain a particular high α-carotene content of Kuroda carrot type. Polyacetylenes have significant weight in variety characterization and the three major polyacetylenes found in carrot root [50] are involved in variety discrimination. Busta et *al*. [58] identified 24 FAD2 (-like) sequences involved in polyacetylenes biosynthesis, scattered on carrot genome. Six functionally characterized genes are co-localized on chromosome four. Co-selection is a probable regulation mechanism which could explain this particular metabolic fingerprint. Specialized metabolites involvement in adaptative response to biotic constraints [59] may also explain the constitutive accumulation of some highlighted compounds. Polyacetylenes and terpenes are involved in pest resistance [60,61]. In fact, polyacetylenes have an effective antibacterial [62] and antifungal activity [63] whereas the highlighted terpene β-pinene, exhibits antimicrobial activity [64]. Particular metabolic pattern with high content in polyacetylenes and β-pinene may confer tolerance propensity to biotic of abiotic stress, as shown for Brasilia varieties [65], and explain their role in variety discrimination. Polyacetylene compounds and β-pinene seem to be under strong genetic determinism and constitutively expressed for several carrot varieties. Le Clerc et *al*. [33] identified metabolites QTLs involving β-pinene, falcarindiol and falcarindiol acetate accumulation which actually colocalize with resistance QTLs against *Alternaria dauci* on chromosome four, consistently with Busta et *al*. [58] who found one FAD2 and five FAD2-like genes on the same chromosome portion. It suggests that these genes are directly involved in polyacetylene accumulation and *in fine* pest resistance. Nevertheless, Keilwagen et *al*. [66] highlighted β-pinene accumulation QTL on other chromosomes. Contrary to falcarindiol compounds accumulation determinism, β-pinene accumulation seems to involve other, more indirect, regulation mechanisms.

Nevertheless, the highlighted compounds linked to stress tolerance such as polyacetylenes and β-pinene are also bitterness related [33]. A balance between pest resistance ability compounds and taste related compound must be considered to valuate carrot potential without compromising taste. Other highlighted compounds in variety discrimination present sensorial properties as (Z)-α-bisabolene which relates to earthy taste [67], whereas citrate is related to sour taste and may inhibit sucrose perception by taste receptor cells [68]. Polyphenol compounds are largely involved in variety discrimination but remain mostly unknown, deserving a more comprehensive study. Finally, carbohydrate and β-carotene content are not discriminating, probably due to general improvement through breeding for these major quality traits.

### Evaluation of varietal metabolite accumulation potential considering environmental sensitivity of variety

More influenced by environment, compounds were characterized for the differential of phenotypic plasticity between varieties. Propensity for metabolite accumulation was evaluated thanks to environmental sensitivity of varieties corresponding to the dynamic stability concept [40]. This latter concept considers environmental effect in addition to varietal effect and brings to light varietal behaviour across numerous environments with a prediction purpose for variety. A higher differential plasticity was observed mainly for terpenes molecules and phenolic compound. D-germacrene, myristicin and 6-methoxymellein are among compounds with the highest plasticity differential. As shown by AMMI results, these 2 latter compounds seem to have similar pattern with common locations and varieties involved in accumulation, suggesting a co-regulation mechanism in their accumulation. 6-methoxymellein is involved in partial resistance of carrot to diseases [69] and myristicin is recognized thanks to its anti-fungal activity [70]. In addition, myristicin seems to potentiate the action of phytoalexins as 6-methoxymellein in carrot root [71]. Kramer et *al*. (2010) [72] showed that 6-methoxymellein and myristicin levels were both enhanced after ethylene treatment, a phytohormone involved in biotic and abiotic stress [73]. D-germacrene plays a leading part in interaction of plant with environment and has a key role in sesquiterpene biosynthesis as precursor of numerous sesquiterpenes [74]. Contrary to previous compounds, it is involved in plant herbivore defense [75,76] and enhanced by methyl jasmonate phytohormone [77]. Several studies highlight D-germacrene enhancement in case of several abiotic disturbances as water deficit stress for specific drought tolerant thyme species compared to sensitive species [78], UV-B irradiance [79] and cold treatment [80]. A putative common up-regulation mechanism seems to involve D-germacrene synthase in case of different climate change related factors (heat, cold, ozone) [81]. D-germacrene ability to exhibit powerful antioxidant activity thanks to its extra cyclic methylene moiety [82] can help to struggle against the deleterious effects of free radicals and ROS resulting from some abiotic stress. Thus, high specialized metabolite content may characterize a response to biotic or abiotic stress [83] and promote acclimation potential of plants. As shown by Hawes et *al*. (2007) in their works on *Halozetes belgicae (Michael)* [84], plasticity and superplasticity play a leading part on acclimation potential and plasticity patterns are related to ecological niches repartition of phenotypes studied. Following this reasoning, high phenotypic plasticity for compounds related to disease resistance or abiotic stress tolerance could be a potential way to enhance acclimation potential of varieties.

Globally, plants recognize environmental signals, as well as abiotic [85,86] or biotic [87] signals via sensors. Differential of environmental sensitivity between varieties may be explained either at signal acquisition level, with more sensitive receptor, or signal transduction level, with an increase of transcription signal [88]. In their review, Des Marais et *al*. (2013) [89] draw up a non-exhaustive list of genes underlying genotype-by-environment interaction in plant abiotic traits and bring to light a majority of transcription factors, photoreceptors and in less extent enzyme, binding protein and 2-component signaller. Likewise, transcription factors are largely involved in plant response to biotic stress [90,91]. Several recent studies stress out the transcription factor involvement in stress tolerance and their potential for crop improvement [92,93]. The study of transcription factors activity would be necessary to better understand the differences of varietal environmental sensitivity for terpenes and phenolic compounds underlined in our study. Since transposable elements are known to be involved in environmental adaptation [94], their study would help understand the underlying mechanisms of plasticity capacity. Interestingly, phenotypic plasticity is more important for exotic carrot types than Nantes carrot types and they react in different way to the environment compared to the Nantes carrot types. These genetic backgrounds could be an interesting resource for breeding perspective.

Our study has deepened six compounds of interest for which varieties have substantial different level of phenotypic plasticity. Depending on the compound, several strategies may be considered to improve the content: a breeding effort could be envisaged to reach the expected level as for D-germacrene, breeding could target specific classes of environments as for myristicin and 6-methoxymellein, or cultivating a variety on propitious environment where it expresses best its potential. Ensuring the highest or lowest content, according to positive or negative effect of a compound on quality, for a variety with specific adaptability requires a better knowledge of the critical environmental factors involved in accumulation.

## Conclusions

Metabolic profiling occurs as an efficient prospective approach in vegetable quality elaboration to valuate whole varietal potential, guarantee crop nutritional and resistance ability without compromising taste. This tool used in conjunction with large carrot diversity and contrasted cropping locations allows to grasp phenotypic variability and evaluate determinant factor involved on a wide range of metabolite content simultaneously. Two opposites concept at first sight, stability and environmental sensitivity, were considered in a complementary way in order to find the most suitable biochemical compounds to be selected for a breeding approach and vegetable quality improvement perspective. Our work led to the identification of compounds to target in priority, for production and breeding purposes in carrot, with adapted strategies depending on genotype-environment interaction level. Thus, variety plasticity needs to be better considered in vegetable crops and regarding quality traits, in order to raise production value and meet consumer demand. Transposable to other vegetables, this approach coupling metabolic profiling to stability and environmental sensitivity analysis offers perspectives for vegetable production improvement. Further work is underway to establish links between nutritional and sensory quality on one hand and identification of agroclimatic factors involved in key metabolites content variation on the other hand.

## Supporting information

S1 Fig Confusion matrix between Random forest classification algorithm prediction and true class. True class in line and predicted class in column. Diagonal represents proportion of individuals well ranked. Dark color represents the totality of well ranked individuals while a lighter color symbolizes a less important proportion of well predicted individuals.
(TIF)

S1 Table Description of cropping location. (DOCX)

S2 Table Variety content in discriminating compounds used in Fig 4 heatmap. *x*: *Non applicable*. CIT is expressed in grams per 100g fresh weight, polyacetylenes (FaDOH, FaDoAc and FaOH) are expressed in ppm 4-chlrorobenzophenone, volatiles compounds (T2, S1, S6 and S10) are expressed in ppb 2-octanol equivalent, acar is expressed in milligrams per 100g fresh weight and polyphenols (P…) are expressed in nanograms per g fresh weight.
(DOCX)

S3 Table Variety variation data for six selected compounds on 20 environments. (DOCX)

S4 Table Mass spectrometric information about polyphenols detected in orange carrot, in the negative ion mode. (DOCX)
