# Supplementary figures and images for: Multisite evaluation of phenotypic plasticity for specialized metabolites, some involved in carrot quality and disease resistance
Source: PLoS One. 2021 Apr 2;16(4):e0249613. doi: 10.1371/journal.pone.0249613 (PMC8018645; doi:10.1371/journal.pone.0249613)

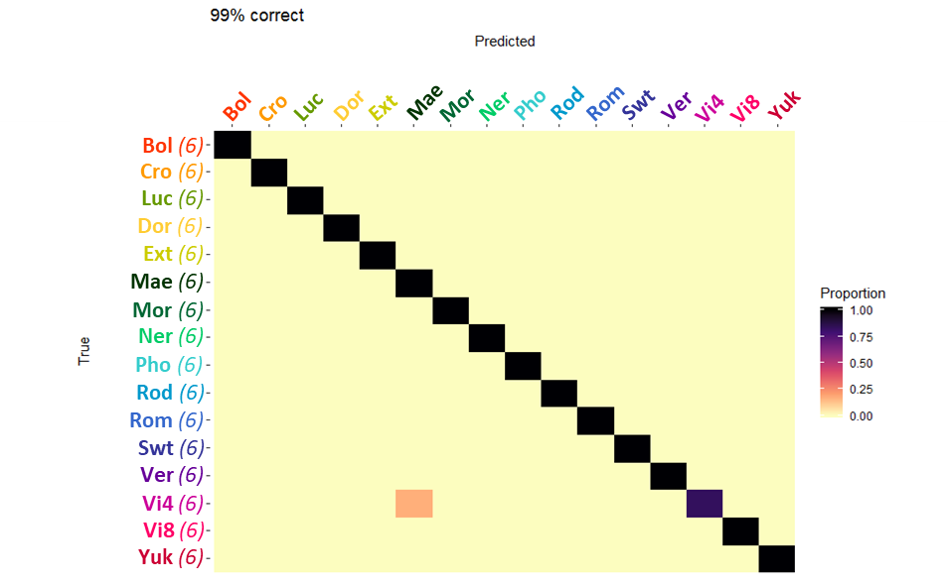

Supplement: S1 Fig — True class in line and predicted class in column. Diagonal represents proportion of individuals well ranked. Dark color represents the totality of well ranked individuals while a lighter color symbolizes a less important proportion of well predicted individuals. (TIF) [file pone.0249613.s001.tif]
