# Supplementary material for: Multisite evaluation of phenotypic plasticity for specialized metabolites, some involved in carrot quality and disease resistance
Source: PLoS One. 2021 Apr 2;16(4):e0249613. doi: 10.1371/journal.pone.0249613 (PMC8018645; doi:10.1371/journal.pone.0249613)
Supplement: S1 Table — (DOCX) [file pone.0249613.s002.docx]

Supporting information Table 1: Description of growing locations

| **Locations** | **Year** | **Code** | **Geographical France area** | **GPS coordinates** | **Crop cycle** | **Temperature mean (°C)** | **Humidity mean (%)** | | **Rainfall sum (mm)** |
| --- | --- | --- | --- | --- | --- | --- | --- | --- | --- |
| Angers (field) | 2016 | Ac6 | Center-West | 47°28'50.3"N 0°36'29.2"W | 125 | 18.70 | | 71.22 | 101 |
| Trensacq | 2016 | Lv6 | South-West | 44°19'14.1"N 0°54'33.6"W | 125 | 19.00 | | 77.61 | 178.5 |
| Aramon | 2016 | Sa6 | South-East | 43°53'25.2"N 4°40'49.8"W | 125 | 22.30 | | 63.01 | 119 |
| Douvres-la-délivrande | 2017 | Mc7 | North | 49°17'25.5"N 0°24'39.8"W | 132 | 16.33 | | 82.66 | 216 |
| Portbail | 2017 | Mp7 | North | 49°20'36.2"N 1°43'8.9"W | 126 | 16.65 | | 82.81 | 234.5 |
| Angers (field) | 2017 | Ac7 | Center-West | 47°28'50.3"N 0°36'29.2"W | 124 | 18.05 | | 74.82 | 239.8 |
| Angers (tunnel) | 2017 | At7 | Center-West | 47°28'50.3"N 0°36'29.2"W | 125 | NA | | NA | 0 |
| La ménitré | 2017 | Av7 | Center-West | 47°24'19.3"N 0°16'33.4"W | 118 | 19.03 | | 70.11 | 187.5 |
| Trensacq | 2017 | Lv7 | South-West | 44°19'14.1"N 0°54'33.6"W | 125 | 16.7 | | 77.93 | 236.5 |
| Ychoux | 2017 | Ly7 | South-West | 44°19'14.1"N 0°54'33.6"W | 126 | 19.03 | | 74.97 | 347 |
| Aramon | 2017 | Sa7 | South-East | 43°53'25.2"N 4°40'49.8"W | 125 | 20.06 | | 63.77 | 69 |
| Cadenet | 2017 | Sc7 | South-East | 43°43'58.3"N 5°22'0.6"W | 130 | 17.45 | | 65.07 | 74.5 |
| La costières | 2017 | Sv7 | South-East | 43°47'58.9"N 4°22'19.8"W | 126 | 18.62 | | 65.07 | 85 |
| Douvres-la-délivrande | 2018 | Mc8 | North | 49°17'25.5"N 0°24'39.8"W | 120 | 17.19 | | 77.32 | 192 |
| Portbail | 2018 | Mp8 | North | 49°20'36.2"N 1°43'8.9"W | 117 | 17.22 | | 85.03 | 171.7 |
| Angers (field) | 2018 | Ac8 | Center-West | 47°28'50.3"N 0°36'29.2"W | 120 | 19.49 | | 69.84 | 158.5 |
| Angers (tunnel) | 2018 | At8 | Center-West | 47°28'50.3"N 0°36'29.2"W | 117 | 22.9 | | 67.12 | 0 |
| La ménitré | 2018 | Av8 | Center-West | 47°24'19.3"N 0°16'33.4"W | 117 | 19.68 | | 64.11 | 111.5 |
| Cestas | 2018 | Lc8 | South-West | 44°44'24"N 0°41'11.2"W | 123 | 17.72 | | 73.93 | 157.1 |
| Trensacq | 2018 | Lv8 | South-West | 44°19'14.1"N 0°54'33.6"W | 123 | 17.12 | | 76.51 | 226.9 |
| Aramon | 2018 | Sa8 | South-East | 43°53'25.2"N 4°40'49.8"W | 120 | 23.57 | | 58.39 | 204 |
| Cadenet | 2018 | Sc8 | South-East | 43°43'58.3"N 5°22'0.6"W | 120 | 21.93 | | 63.97 | 298.5 |
| La costières | 2018 | Sv8 | South-East | 43°47'58.9"N 4°22'19.8"W | 118 | 20.28 | | 66.64 | 353 |
