# Supplementary material for: Multisite evaluation of phenotypic plasticity for specialized metabolites, some involved in carrot quality and disease resistance
Source: PLoS One. 2021 Apr 2;16(4):e0249613. doi: 10.1371/journal.pone.0249613 (PMC8018645; doi:10.1371/journal.pone.0249613)
Supplement: S2 Table — x: Non applicable. CIT is expressed in grams per 100g fresh weight, polyacetylenes (FaDOH, FaDoAc and FaOH) are expressed in ppm 4-chlrorobenzophenone, volatiles compounds (T2, S1, S6 and S10) are expressed in ppb 2-octanol equivalent, acar is expressed in milligrams per 100g fresh weight and polyphenols (P…) are expressed in nanograms per g fresh weight. (DOCX) [file pone.0249613.s003.docx]

Supporting information Table 2: Mean and coefficient of variation of varietal marker used in heatmap (Fig. 4)

| Varieties | CIT | | FaDOH | | FaDOAc | | FaOH | | T2 | | S1 | | S6 | | S10 | | acar | | P2.95 | |
| --- | --- | --- | --- | --- | --- | --- | --- | --- | --- | --- | --- | --- | --- | --- | --- | --- | --- | --- | --- | --- |
|  | mean | *CV* | mean | *CV* | mean | *CV* | mean | *CV* | mean | *CV* | mean | *CV* | mean | *CV* | mean | *CV* | mean | *CV* | mean | *CV* |
| Bol | 0.07 | *11.26* | 5.59 | *14.68* | 0.75 | *14.29* | 4.80 | *18.51* | 497.76 | *38.25* | 0.00 | *x* | 415.22 | *5.26* | 3292.05 | *12.60* | 5.86 | *8.94* | 94.73 | *14.62* |
| Cro | 0.07 | *8.47* | 5.09 | *7.28* | 1.13 | *14.67* | 4.95 | *8.84* | 713.02 | *49.12* | 455.09 | *22.20* | 840.37 | *18.65* | 1761.87 | *21.50* | 8.05 | *9.16* | 77.28 | *42.49* |
| Luc | 0.12 | *12.23* | 4.68 | *25.85* | 0.97 | *9.45* | 3.99 | *18.17* | 632.06 | *4.05* | 17.58 | *118.38* | 1201.98 | *93.39* | 4025.55 | *16.44* | 10.04 | *8.56* | 54.70 | *25.01* |
| Dor | 0.04 | *11.42* | 1.97 | *25.82* | 0.91 | *26.08* | 2.26 | *23.62* | 349.69 | *24.57* | 188.33 | *92.17* | 157.09 | *5.16* | 94.81 | *63.56* | 5.74 | *21.09* | 38.71 | *47.71* |
| Ext | 0.03 | *30.58* | 4.40 | *18.00* | 0.96 | *10.57* | 7.92 | *23.15* | 275.86 | *20.55* | 0.00 | *x* | 923.14 | *47.80* | 238.08 | *63.90* | 9.49 | *5.95* | 15.42 | *94.76* |
| Mae | 0.06 | *27.75* | 3.06 | *16.57* | 1.00 | *3.07* | 2.84 | *9.59* | 806.78 | *79.23* | 349.80 | *17.03* | 1511.22 | *14.08* | 1796.35 | *33.78* | 5.36 | *4.99* | 49.29 | *101.17* |
| Mor | 0.04 | *13.47* | 4.28 | *7.32* | 1.14 | *7.72* | 2.07 | *20.07* | 1816.09 | *14.01* | 1283.79 | *14.23* | 857.44 | *32.78* | 2473.28 | *43.51* | 5.57 | *1.73* | 161.39 | *11.36* |
| Ner | 0.03 | *23.44* | 4.83 | *8.71* | 1.50 | *9.93* | 2.29 | *12.75* | 516.20 | *37.09* | 3.38 | *173.21* | 334.22 | *5.18* | 311.68 | *114.86* | 5.73 | *17.54* | 244.24 | *16.63* |
| Pho | 0.06 | *14.00* | 4.30 | *8.58* | 0.74 | *7.12* | 3.16 | *24.59* | 616.45 | *8.02* | 108.41 | *89.81* | 212.48 | *43.45* | 1331.89 | *43.15* | 6.99 | *4.44* | 17.11 | *23.75* |
| Rod | 0.06 | *22.49* | 5.85 | *28.62* | 1.03 | *28.88* | 3.61 | *45.51* | 375.45 | *26.04* | 128.05 | *20.25* | 527.54 | *33.18* | 8381.00 | *18.53* | 7.44 | *33.90* | 98.16 | *32.77* |
| Rom | 0.04 | *19.10* | 2.53 | *21.73* | 0.60 | *6.97* | 2.83 | *16.28* | 410.79 | *25.57* | 704.56 | *18.13* | 255.48 | *29.89* | 2534.54 | *35.74* | 7.47 | *10.15* | 140.51 | *12.73* |
| Swt | 0.03 | *7.98* | 2.57 | *23.52* | 0.79 | *4.83* | 3.19 | *15.42* | 271.04 | *18.45* | 0.00 | *x* | 1515.47 | *43.17* | 2110.10 | *15.96* | 10.29 | *7.05* | 0.42 | *173.21* |
| Ver | 0.04 | *2.13* | 8.40 | *8.77* | 1.24 | *17.53* | 6.09 | *35.79* | 4370.64 | *11.98* | 1.37 | *173.21* | 639.31 | *51.19* | 3762.07 | *22.15* | 6.36 | *36.81* | 28.98 | *27.62* |
| Vi4 | 0.07 | *40.78* | 4.22 | *20.62* | 1.26 | *15.92* | 6.37 | *2.66* | 690.77 | *19.77* | 1238.41 | *28.27* | 602.95 | *36.23* | 4573.49 | *18.20* | 5.50 | *15.75* | 11.52 | *99.16* |
| Vi8 | 0.08 | *45.00* | 8.84 | *5.21* | 2.18 | *6.25* | 4.63 | *23.50* | 614.64 | *28.94* | 3.42 | *173.21* | 3071.08 | *40.26* | 133.61 | *58.62* | 14.07 | *11.26* | 17.12 | *64.39* |
| Yuk | 0.05 | *4.74* | 3.32 | *28.01* | 0.52 | *14.93* | 2.68 | *16.10* | 2152.33 | *31.32* | 0.00 | *x* | 770.29 | *21.35* | 566.40 | *54.69* | 5.96 | *28.36* | 94.69 | *49.98* |

| Varieties | P3.47 | | P4.41 | | P4.91 | | P5.08 | | P5.13 | | P5.85 | | P5.97 | | P6.05.2 | | P7.16 | | P8.51 | |
| --- | --- | --- | --- | --- | --- | --- | --- | --- | --- | --- | --- | --- | --- | --- | --- | --- | --- | --- | --- | --- |
|  | mean | *CV* | mean | *CV* | mean | *CV* | mean | *CV* | mean | *CV* | mean | *CV* | mean | *CV* | mean | *CV* | mean | *CV* | mean | *CV* |
| Bol | 219.72 | *29.14* | 0.80 | *173.21* | 0.00 | *x* | 0.00 | *x* | 0.00 | *x* | 87.00 | *27.59* | 60.86 | *33.50* | 0.00 | *x* | 0.00 | *x* | **126.91** | *35.26* |
| Cro | 133.34 | *18.69* | 0.33 | *173.21* | **10.71** | *40.69* | 1029.48 | *16.37* | 53.19 | *13.75* | 198.51 | *8.95* | 65.46 | *28.41* | 0.00 | *x* | 0.00 | *x* | 0.57 | *173.21* |
| Luc | 174.47 | *20.01* | 0.00 | *x* | 0.00 | *x* | 945.43 | *13.18* | 0.00 | *x* | 129.16 | *45.63* | 96.63 | *23.12* | 0.00 | *x* | 0.00 | *x* | 23.65 | *31.17* |
| Dor | 122.28 | *24.25* | 6.91 | *158.99* | 0.00 | *x* | 937.75 | *18.51* | 0.00 | *x* | 93.30 | *16.78* | 28.61 | *44.06* | 0.00 | *x* | 0.00 | *x* | 0.00 | *x* |
| Ext | 21.58 | *51.52* | 9.40 | *115.03* | 0.72 | *123.41* | 17.64 | *106.33* | 0.00 | *x* | 132.13 | *10.38* | 51.08 | *27.27* | 3.81 | *93.85* | 0.00 | *x* | 0.00 | *x* |
| Mae | 133.64 | *27.02* | 90.89 | *9.79* | 0.00 | *x* | 545.47 | *32.71* | 0.00 | *x* | 115.44 | *29.23* | 20.12 | *16.90* | 0.00 | *x* | 0.00 | *x* | 4.63 | *173.21* |
| Mor | 119.98 | *31.62* | 3.99 | *87.40* | 0.29 | *173.21* | 624.12 | *10.19* | 2.49 | *173.21* | 144.90 | *11.62* | 33.56 | *22.69* | 0.00 | *x* | 0.00 | *x* | 48.10 | *62.67* |
| Ner | 134.76 | *10.61* | 0.00 | *x* | 0.00 | *x* | 662.83 | *12.06* | 0.00 | *x* | 0.57 | *134.13* | 3.82 | *113.85* | 4.95 | *43.98* | 0.00 | *x* | 0.00 | *x* |
| Pho | 72.54 | *26.17* | 0.00 | *x* | 0.00 | *x* | 496.73 | *11.17* | 0.00 | *x* | 15.82 | *90.06* | 74.61 | *19.69* | 0.00 | *x* | 0.00 | *x* | 1.51 | *121.71* |
| Rod | 112.60 | *30.27* | 3.55 | *173.21* | 4.79 | *90.86* | 110.51 | *43.19* | 0.00 | *x* | 38.49 | *68.62* | 35.95 | *69.70* | 20.00 | *75.28* | 0.00 | *x* | 17.25 | *53.25* |
| Rom | 261.79 | *33.54* | 37.46 | *145.29* | 0.00 | *x* | 643.32 | *19.62* | 0.00 | *x* | 270.00 | *23.00* | 37.52 | *66.16* | 0.00 | *x* | 0.00 | *x* | 37.70 | *5.99* |
| Swt | 18.78 | *103.24* | 0.00 | *x* | 0.00 | *x* | 0.00 | *x* | **97.47** | *3.00* | **349.05** | *23.58* | 86.26 | *22.04* | **56.20** | *58.14* | 0.00 | *x* | 1.17 | *173.21* |
| Ver | 117.70 | *26.55* | 28.01 | *77.69* | 0.00 | *x* | 694.17 | *16.62* | 1.37 | *173.21* | 95.69 | *21.06* | 51.63 | *17.37* | 0.00 | *x* | 0.00 | *x* | 0.00 | *x* |
| Vi4 | 141.98 | *20.02* | 45.89 | *104.12* | 0.00 | *x* | 879.46 | *41.66* | 0.00 | *x* | 127.42 | *19.31* | 13.50 | *127.86* | 0.00 | *x* | 0.00 | *x* | 59.15 | *47.76* |
| Vi8 | 169.98 | *27.85* | **146.90** | *44.07* | 5.75 | *86.86* | 636.11 | *29.68* | 11.27 | *30.94* | 320.19 | *26.44* | 145.11 | *29.68* | 0.00 | *x* | 0.00 | *x* | 0.00 | *x* |
| Yuk | **419.63** | *22.66* | 105.86 | *58.02* | 0.00 | *x* | 1040.23 | *9.11* | 0.00 | *x* | 210.75 | *20.08* | 37.32 | *41.88* | 0.00 | *x* | **69.64** | *8.02* | 0.00 | *x* |
