# Supplementary material for: Multisite evaluation of phenotypic plasticity for specialized metabolites, some involved in carrot quality and disease resistance
Source: PLoS One. 2021 Apr 2;16(4):e0249613. doi: 10.1371/journal.pone.0249613 (PMC8018645; doi:10.1371/journal.pone.0249613)
Supplement: S3 Table — (DOCX) [file pone.0249613.s004.docx]

Supporting information Table 3: Variety data for selected compounds on 20 environments

| **Compounds** | **Varieties** | **Varietal mean** | **bi** | **Ecovalence** | **Ecovalence (%)** |
| --- | --- | --- | --- | --- | --- |
| **PP2**  (A) | Cro | 675.151 ^c^ | 0.210 *** | 8.64E+07 | 10.674 |
|  | Dor | 695.539 ^c^ | 0.153 *** | 9.51E+07 | 11.750 |
|  | Vi8 | 2394.956 ^b^ | 0.941 | 2.90E+07 | 3.584 |
|  | Mae | 5221.178 ^a^ | 1.417 . | 1.56E+08 | 19.256 |
|  | Ver | 6607.773 ^a^ | 2.279 *** | 4.43E+08 | 54.735 |
| **6MM**  (B) | Cro | 0.799 ^c^ | 0.364 *** | 8.28E+01 | 35.456 |
|  | Vi8 | 1.126 ^c^ | 0.726 *** | 2.59E+01 | 11.105 |
|  | Dor | 2.372 ^b^ | 1.099 * | 7.24E+00 | 3.099 |
|  | Mae | 3.17 ^ab^ | 1.436 *** | 5.35E+01 | 22.901 |
|  | Ver | 3.979 ^a^ | 1.375 ** | 6.41E+01 | 27.439 |
| **S6**  (C) | Dor | 73.013 ^e^ | 0.055 *** | 2.87E+06 | 17.564 |
|  | Ver | 213.697 ^d^ | 0.194 *** | 2.23E+06 | 13.628 |
|  | Cro | 714.432 ^c^ | 0.659 ** | 8.72E+05 | 5.332 |
|  | Mae | 1179.489 ^b^ | 1.770 *** | 3.18E+06 | 19.407 |
|  | Vi8 | 2083.884 ^a^ | 2.322 *** | 7.21E+06 | 44.069 |
| **FaDOAc**  (D) | Dor | 6.898 ^c^ | 1.201 | 5.28E+01 | 18.316 |
|  | Mae | 6.915 ^c^ | 0.644 * | 3.85E+01 | 13.356 |
|  | Ver | 7.64 ^c^ | 0.964 | 2.87E+01 | 9.936 |
|  | Cro | 9.646 ^b^ | 0.624 . | 6.54E+01 | 22.688 |
|  | Vi8 | 16.075 ^a^ | 1.567 * | 1.03E+02 | 35.703 |
| **T2**  (E) | Dor | 303.98 ^c^ | 0.313 *** | 4.63E+06 | 5.823 |
|  | Cro | 420.925 ^b^ | 0.346 *** | 4.27E+06 | 5.369 |
|  | Vi8 | 486.856 ^b^ | 0.347 *** | 4.33E+06 | 5.444 |
|  | Mae | 502.312 ^b^ | 0.422 *** | 3.33E+06 | 4.191 |
|  | Ver | 4767.171 ^a^ | 3.572 *** | 6.30E+07 | 79.173 |
| **acar**  (F) | Ver | 2.845 ^d^ | 0.553 *** | 5.40E+00 | 14.058 |
|  | Mae | 3.019 ^cd^ | 0.643 *** | 4.05E+00 | 10.545 |
|  | Dor | 3.442 ^c^ | 0.808 ** | 1.81E+00 | 4.716 |
|  | Cro | 4.5 ^b^ | 1.082 | 4.66E+00 | 12.128 |
|  | Vi8 | 5.802 ^a^ | 1.913 *** | 2.25E+01 | 58.553 |
