# Supplementary material for: Multisite evaluation of phenotypic plasticity for specialized metabolites, some involved in carrot quality and disease resistance
Source: PLoS One. 2021 Apr 2;16(4):e0249613. doi: 10.1371/journal.pone.0249613 (PMC8018645; doi:10.1371/journal.pone.0249613)
Supplement: S4 Table — (DOCX) [file pone.0249613.s005.docx]

Supporting information Table 4: Mass spectrometric information about polyphenols detected in orange carrot, in the negative ion mode.

| **Code** | **Retention time (min)** | **Compounds** | **Possible molecular formula** | **Mass (m/z)** | **MS² main fragment (m/z)** |
| --- | --- | --- | --- | --- | --- |
| P2.95 | 2.95 | unknown | unknown | 409.0426 | minor peak |
| P3.47 | 3.47 | unknown | C14H18O9 | 329.0895 | 329.0895 (15%), 167.0346 (100%) |
| P4.41 | 4.41 | unknown | C13H16O9 | 315.0715 | 315.0715 (45%), 167.0346 (100%) |
| P4.91 | 4.91 | unknown | C13H24O9 | 323.1353 | 323.1353 (100%), 191.0568 (70%) |
| P5.08 | 5.08 | unknown | C18H24O12 | 431.1213 | 431.1213 (100%), 329.0858 (10%), 125.0234 (45%), 863.2443 [2M-H] (10%) |
| P5.13 | 5.13 | unknown, with sulfate | unknown | 467.1583 | 467.1583 (100%), 96.9601 (50%) |
| P5.85 | 5.85 | unknown, with sulfate | C13H22O6S | 305.1055 | 305.1055 (80%), 96.9601 (100%) |
| P5.97 | 5.97 | unknown, with hexoside | C16H26O9 | 361.1517 | 361.1517 (30%), 199.0984 (60%), 155.1084 (100%) |
| P6.05.2 | 6.05 | unknown | C16H20O11 | 387.0933 | 387.0933 (35%), 249.0604 (30%), 137.0217 (100%) |
| P7.16 | 7.16 | unknown | unknown | 289.111 | minor peak |
| P8.51 | 8.51 | unknown, with hexoside, pentoside | C21H36O10 | 447.2249 | 447.2249 (100%), 315.1803 (20%), 161.0442 (20%), 101.0223 (11%) |
